# Characterization of New Wheat-*Thinopyrum intermedium* Derivative Lines with Superior Genes for Stripe Rust and Powdery Mildew Resistance

**DOI:** 10.3390/plants13162333

**Published:** 2024-08-22

**Authors:** Zhihui Yu, Guangrong Li, Zhiqiang Zheng, Hongjin Wang, Zujun Yang

**Affiliations:** 1 School of Life Science and Technology, University of Electronic Science and Technology of China, Chengdu 610054, China; 201911140605@std.uestc.edu.cn (Z.Y.); ligr28@uestc.edu.cn (G.L.); 202221140503@std.uestc.edu.cn (Z.Z.); 2 College of Life Sciences, Zaozhuang University, Zaozhuang 277100, China

**Keywords:** *Thinopyrum intermedium*, wheat, ND-FISH, molecular marker analysis, disease resistance

## Abstract

The wild species *Thinopyrum intermedium* (genome JJJ^S^J^S^StSt) serves as a valuable germplasm resource providing novel diseases resistance and agronomically important genes for wheat improvement. Two wheat-*Th. intermedium* partial amphiploids, TAI7045 (2n = 56) and 78784 (2n = 56), exhibit high resistance to stripe rust and powdery mildew, and their chromosome constitutions have been characterized. With the aim to transfer novel resistance genes from *Th. intermedium*, the crosses of common wheat line MY11 with TAI7045 and 78784 were produced, and their individual F_2_-F_5_ progenies were characterized using sequential non-denaturing fluorescence in situ hybridization (ND-FISH) and molecular markers. We identified a set of wheat-*Th. intermedium* addition lines, involving the chromosomes 1St-J^S^, 2St, 2St-J^S^, 3St, 4J, 4St, 5St, 5J.St, 6J^S^.J, and 7J^S^. Above all, the stable wheat-*Th. intermedium* small segmental translocation lines with chromosomes 4DS.4DL-4StL-4DL-4JL and 4DS.4DL-4StL-4DL were selected. Combining data from specific marker amplification and resistance evaluation, we mapped the gene(s) for resistance to powdery mildew and stripe rust in the 233.56–329.88 Mb region of the long arm of the 4St chromosome from the reference *Th. intermedium* genome. The new wheat-*Th. intermedium* introgressions will be used as novel germplasm for breeding purposes.

## 1. Introduction

As one of the three most important food crops, common wheat (*Triticum aestivum* L., 2n = 6x = 42, AABBDD) is cultivated widely in the world, providing the staple food source for 30% of the global population [1]. The production of wheat is seriously affected by many diseases such as powdery mildew and rusts. Powdery mildew, caused by *Blumeria graminis* f. sp. *tritici* (*Bgt*), and stripe rust (yellow rust), caused by *Puccinia striiformis* f. sp. *tritici* (*Pst*), are two serious diseases that frequently occur in most wheat-growing regions subject to moderate temperatures and rainy conditions [2,3] and result in a huge loss of wheat yield [4]. The development and cultivation of resistant cultivars is regarded as the most economical, effective and environmentally friendly approach to control these diseases. Although considerable numbers of powdery mildew and stripe rust resistance genes have been identified and used in wheat breeding [5,6], many previously resistant wheat varieties are becoming susceptible due to the continuous appearance of new pathogen races [7,8]. For instance, there are 17 stripe rust resistance genes and 41 powdery mildew resistance genes with the official name originated from wild species of wheat [9]. Among them, genes such as *Pm8*, *Yr9* and *Yr17* have completely or partially lost their effectiveness [10,11]. It is thus essential to discover and exploit novel resistant sources for cultivar improvement via the wide hybridization of wheat with uncultivated species.

The intermedium wheatgrass (previously termed *Agropyron intermedium*) *Thinopyrum intermedium* (Host) Barkworth & D.R. Dewey (2n = 6x = 42, JJJ^S^J^S^StSt) is an allohexaploid perennial species with a native distribution throughout the Mediterranean and Eastern Europe [12]. It possesses many potentially useful agronomic characteristics, such as stress tolerance and disease resistance, that could be used for wheat improvement [13,14]. Because of its high crossability with common wheat, researchers have developed a series of wheat-*Th. intermedium* partial amphiploids and a number of wheat-*Thinopyrum* chromosome addition, substitution and translocation lines in the past few decades [15,16]. Several genes for improving disease resistance and seed quality have been transferred into wheat from *Th. intermedium* through interspecific hybridization [16,17,18,19]. For example, two powdery mildew resistance genes, *Pm40* and *Pm43*, and one stripe rust resistance gene, *Yr50*, which were derived from intermediate wheatgrass, have been mapped to chromosomes 7BS, 2DL and 4BL, respectively [20,21,22]. The introgressed *Th. intermedium* chromosome fragment can be identified and tracked by genomic in situ hybridization (GISH), fluorescence in situ hybridization (FISH), molecular marker analysis, SNP array analysis, etc. [23,24,25].

However, the efficiency of identifying individual *Th. intermedium* chromosomes in a wheat background is relatively low, since it takes a lot of time to assign polyploid alien chromosomes to specific homoeologous linkage groups and sub-genomes. For example, Yu et al. [26] identified two new wheat-*Th. intermedium* addition lines, Hy36 and Hy37, by 350 PLUG markers, 890 CINAU markers and 90K iSelect SNP array, and the results confirmed that the added chromosomes in Hy36 and Hy37 were 5J^S^S.3StS and 5JS.3StS, respectively. Given the complex genomic composition of *Th. intermedium* and numerous chromosomal rearrangements among three sub-genomes, we built a new combination of oligonucleotide probes and Oligo-FISH painting system to improve the identification efficiency of *Th. intermedium* chromosomes [26,27]. Two wheat-*Th. intermedium* partial amphiploids, TAI7045 and 78784, displayed high resistance to several fungal diseases including powdery mildew and stripe rust [18,27]. The objectives of the present study were to develop a set of wheat-*Th. intermedium* introgression lines from TAI7045 and 78784 and map the resistance gene(s) into specific *Th. intermedium* regions, which will potentially be useful for wheat germplasm innovation.

## 2. Results

### 2.1. Comparative Analysis of Karyotype between TAI7045 and 78784

**Figure 1:**
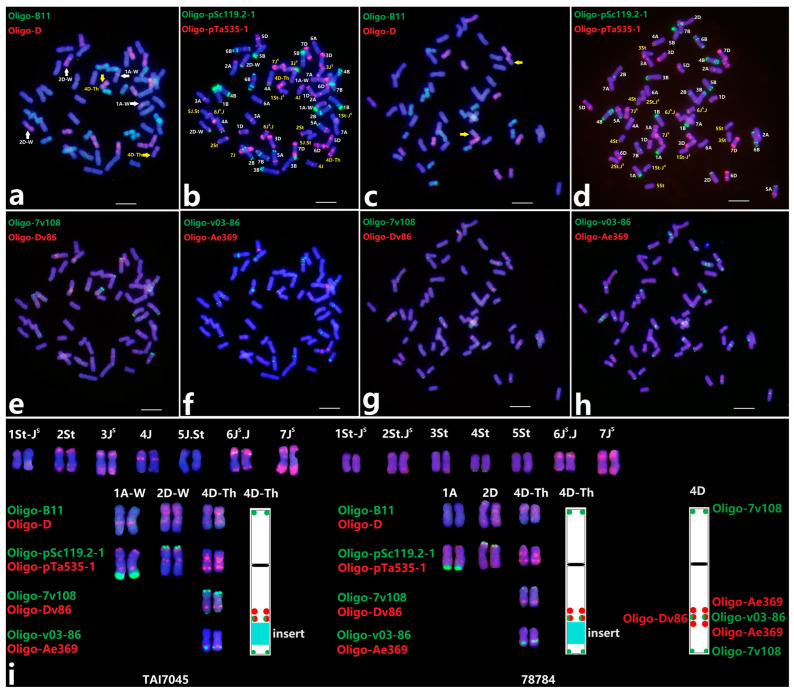
Sequential ND-FISH patterns of wheat-*Th. intermedium* partial amphiploid TAI7045 (**a**,**b**,**e**, **f**) and 78784 (**c**,**d**,**g**,**h**) with multiple probes. The probes Oligo-B11 (green) + Oligo-D (red) (**a**,**c**), Oligo-pSc119.2-1 (green) and Oligo-pTa535-1 (red) (**b**,**d**), Oligo-7v108 (green) + Oligo-Dv86 (red) (**e**,**g**), and Oligo-v03-86 (green) + Oligo-Ae369 (red) (**f**,**h**) are presented, respectively. White arrows indicate the recombinant chromosomes in wheat background, while yellow arrows indicate the translocation chromosomes between wheat and *Th. intermedium*. The *Th. intermedium* chromosomes and recombinant chromosomes of TAI7045 and 78784 are shown (**i**). The period “.” means that the breakpoint of the chromosome translocation events was located in the region of the centromere, and the hyphen “-” means that the breakpoint was located in the other regions. Bars represent 10 μm.

The alien chromosomal constitution of two wheat-*Th. intermedium* partial amphiploids, TAI7045 (2n = 56) and 78784 (2n = 56), has been determined by the bulked Oligo-based FISH painting and ND-FISH in our previous study [27]. Seven pairs of *Th. intermedium* chromosomes of TAI7045 and 78784 can be identified using probes Oligo-B11, Oligo-pSc119.2-1 and Oligo-pTa535-1, the former as linkage groups 1St-J^S^, 2St, 3J^S^, 4J, 5J.St, 6J^S^.J and 7J^S^ and the latter as linkage groups 1St-J^S^, 2St.J^S^, 3St, 4St, 5St, 6J^S^.J and 7J^S^ (Figure 1). The chromosomes 1St-J^S^ and 5J.St from TAI7045 and the chromosomes 1St-J^S^, 3St and 5St from 78784 can be distinguished easily with the probes Oligo-pTa71-2 and Oligo-pSc200 (Figure S1), and two 1St-J^S^ chromosomes displayed different green bands labeled with Oligo-pSc200.

Strikingly, we also observed a pair of identical wheat-*Th. intermedium* intercalary translocation chromosomes in TAI7045 and 78784, tentatively named 4D-Th. The introgressed *Th. intermedium* chromosome segments were identified using the probe Oligo-B11, and it produced signals on the sub-telomeric regions in the long arm of 4D-Th (Figure 1a,i). Four newly developed probes, Oligo-7v108, Oligo-Dv86, Oligo-v03-86 and Oligo-Ae369, could be used for detecting different regions of the 4D chromosomes, particularly on its long arm (Figure S2 and Figure 1i). Probe Oligo-Ae369 could generate two adjacent strong signals on the 4DL; however, the long arms of 4D-Th chromosomes only showed one distinct Oligo-Ae369 signal away from the end. It is noteworthy that the 4D-Th chromosomes retained a weak Oligo-7v108 signal on the end of its long arm, which indicated the interstitial deficiency of 4DL. In addition, two visible differences in wheat chromosomes between TAI7045 and 78784 were detected with the probe Oligo-D, and four varied wheat chromosomes 1A-W and 2D-W in TAI7045 are shown in Figure 1i. One small D-genome chromosome segment was translocated interstitially into in the 1AL, and the recombinant chromosome 2D-W displayed a large deletion on 2DL.

### 2.2. Transmission of Thinopyrum Chromosomes and Identification of Stable Wheat-Th. intermedium-Derived Lines

**Figure 2:**
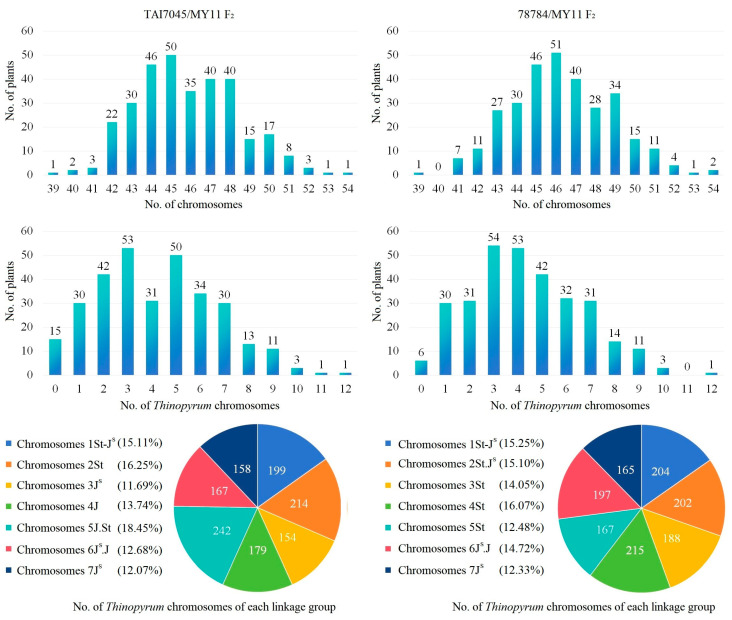
Number of *Thinopyrum* chromosomes in F_2_ generation of two crosses, TAI7045/MY11 (**left**) and 78784/MY11 (**right**).

The combination of the four probes Oligo-pSc119.2-1, Oligo-pTa535-1, Oligo-pTa71-2 and Oligo-pSc200 was applied to identify wheat and *Th. intermedium* chromosomes of individual plants from TAI7045- and 78784-derived progenies (Figure S3). A total of 314 F_2_ plants from the cross TAI7045/MY11 and 308 F_2_ plants from the cross 78784/MY11 were karyotypically analyzed using ND-FISH. The chromosomal numbers of two F_2_ populations were distributed from 39 to 54 (Figure 2). Plants possessing chromosome numbers less than 42 may be due to the abnormal meiotic behavior of wheat chromosomes affected by *Thinopyrum* chromatin introgression. In F_2_, the chromosomes 5J.St from TAI7045 and the chromosomes 4St from 78784 showed the highest transmission frequencies, 18.45% and 16.07%, in seven linkage groups, respectively. Chromosomes 7J^S^ with relatively low transmission frequencies (12.07% and 12.33%) were also observed in the two F_2_ populations.

Meanwhile, a total of 72 and 61 F_2_ progenies were selected from the hybrids of TAI7045/MY11 and 78784/MY11, respectively. The seeds carrying one or two alien chromosomes were performed for karyotypic analysis using ND-FISH. In the F_3_ of TAI7045/MY11, except for 3J^S^ chromosomes, the wheat-*Th. intermedium* 1St-J^S^, 2St, 6J^S^.J and 7J^S^ disomic addition lines and 4J and 5J.St monosomic addition lines were successfully obtained (Figure S4). The wheat-*Th. intermedium* 1St-J^S^, 2St-J^S^, 3St, 4St and 6J^S^.J disomic addition lines and 5St and 7J^S^ monosomic addition lines were also gained from the F_3_ populations of the cross 78784/MY11 (Figure S5). These novel wheat-*Th. intermedium*-derived lines are expected to benefit the genetic resource research and development of *Th. intermedium*. Moreover, we found some wheat-wheat chromosomal rearrangement events in the F_3_, such as 4BS.4AL and 4AS.4BL (Figure S5f,h), 3BS.5AS and 3BL.5AL (Figure S5j), 5BS.7BS and 5BL.7BL translocation chromosomes (Figure S5k), which may be caused by the introduction of *Th. intermedium* chromosomes in the wheat background.

### 2.3. ND-FISH of Two Distinct Wheat-Th. intermedium Translocation Lines by Multiple Oligo Probes

**Figure 3:**
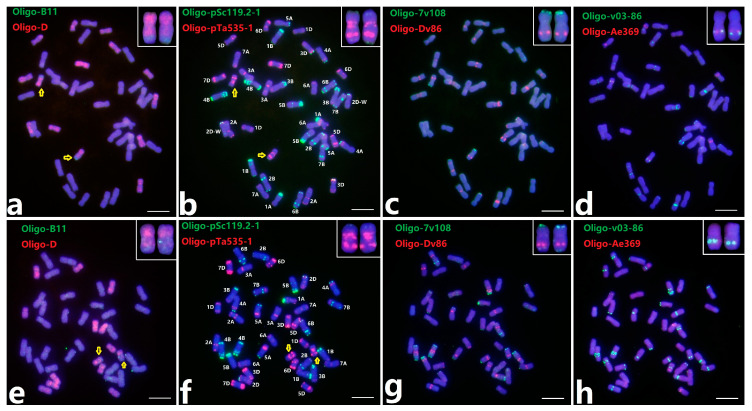
Sequential ND-FISH of the lines WT4D-1 (**a**–**d**) and WT4D-2 (**e**–**h**) by multiple probes. The probes Oligo-B11 (green) + Oligo-D (red) (**a**,**e**), Oligo-pSc119.2-1 (green) + Oligo-pTa535-1 (red) (**b**,**f**), Oligo-7v108 (green) + Oligo-Dv86 (red) (**c**,**g**), and Oligo-v03-86 (green) + Oligo-Ae369 (red) (**d**,**h**). The wheat-*Th. intermedium* translocation chromosomes are shown by arrows and the cut-and-paste chromosomes at the top right. Bars represent 10 μm.

In addition to the above-mentioned wheat-*Th. intermedium* addition lines, we also identified two distinct wheat-*Th. intermedium* translocation lines WT4D-1 and WT4D-2 in F_3_ to F_5_ from the crosses TAI7045/MY11 and 78784/MY11, respectively. Sequential multi-color ND-FISH with eight probes, Oligo-B11, Oligo-D, Oligo-pSc119.2-1, Oligo-pTa535-1, Oligo-7v108, Oligo-Dv86, Oligo-v03-86 and Oligo-Ae369, was conducted to characterize the cytologically stable lines WT4D-1 and WT4D-2 (Figure 3). Probes Oligo-B11 and Oligo-D showed the occurrence of two translocation chromosomes between the *Th. intermedium* genomes and D-genome, and probes Oligo-pSc119.2-1 and Oligo-pTa535-1 further revealed the breakpoints located in the long arms of 4D chromosomes. Compared with the 4D chromosomes, the chromosomes WT4D-1 added weak Oligo-pSc119.2-1 signals on the telomeres of the long arm, while the chromosomes WT4D-2 reserved Oligo-7v108 signals located on the end of 4DL (Figure S1 and Figure 3b,g). The chromosomes WT4D-1 and WT4D-2 only carried one FISH pattern of Oligo-Ae369, which indicated the partial deletion of 4DL. Based on the cytogenetic analysis of the lines WT4D-1 and WT4D-2 and their respective parents, we posit that the chromosomes WT4D-1 might derive from the recombination between 4D-Th and 4J in TAI7045, while the chromosomes WT4D-2 were the same as the 4D-Th of 78784 in FISH patterns.

### 2.4. Molecular Marker Analysis for Structure of Chromosomes WT4D-1 and WT4D-2

**Figure 4:**
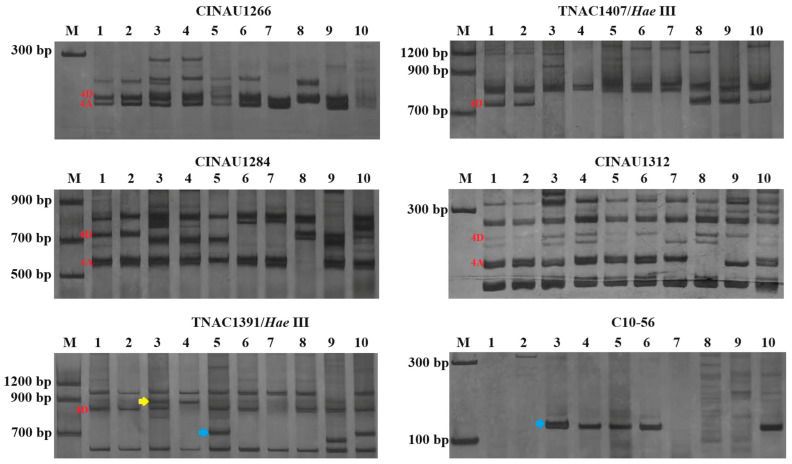
PCR profiling of molecular markers in wheat-*Th. intermedium* lines. M, molecular marker; lane 1: a wheat cultivar Chinese Spring (CS); lane 2: MY11; lane 3: TAI7045; lane 4: WT4D-1; lane 5: 78784; lane 6: WT4D-2; lane 7: nullisomic-4D tetrasomic-4B of CS; lane 8: nullisomic-4A tetrasomic-4D of CS; lane 9: X24C10 (4J/4B substitution line); lane 10: WT78-4 (4St addition line). The chromosome 4A- and 4D-specific bands are indicated in red, and the yellow arrows and blue arrows indicate the chromosome 4J-specific bands and chromosome 4St-specific bands, respectively.

**Figure 5:**
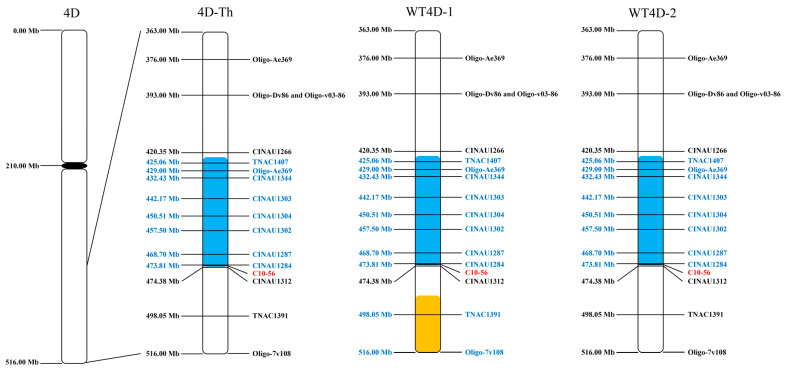
Physical map of the translocation chromosomes 4D-Th, WT4D-1 and WT4D-2 in comparison with 4D chromosomes. The blue molecular markers and Oligo probes indicate the deletion of 4D chromosome fragments. The 4St-specific marker C10-56 indicates the putative breakpoint of the translocation. The chromosome fragments with blue and orange backgrounds represent the introgression of chromosomes 4St and 4J, respectively.

In order to map the breakage-fusion structure of the chromosomes 4D-Th in TAI7045 and 78784, as well as the translocated chromosomes in lines WT4D-1 and WT4D-2 (Figure 4), 60 PLUG markers of wheat homoeologous group 4 specific [28], 89 CINAU markers derived from *Dasypyrum villosum* chromosome 4VL [29], and 58 Thi-specific markers from *Th. intermedium* 4St and/or 4J chromosomes [30] were used with their corresponding location blasted on the updated reference genomes of wheat (https://urgi.versailles.inra.fr/download/iwgsc/IWGSC_RefSeq_Assemblies/v2.0/, accessed on 12 April 2024) and *Thinopyrum intermedium* V3.1 (https://phytozome-next.jgi.doe.gov/info/Tintermedium_v3_1, accessed on 12 April 2024), respectively. The results showed that an intercalary deletion (flanking TNAC1407-CINAU1284) occurred in the chromosomes 4DL in lines 7045, 78784, WT4D-1 and WT4D-2 (Figure 4). The BLAST search showed that the proximal breakpoint may be located in the region of 420.35–425.06 Mb, while the distal breakpoint is in the region of 473.81–474.38 Mb of 4D (Figure 5). Compared to the chromosomes 4D-Th and WT4D-2, the chromosome WT4D-1 still presented a small terminal deficiency (covered TNAC1391 and Oligo-7v108) in the end of 4DL.

**Figure 6:**
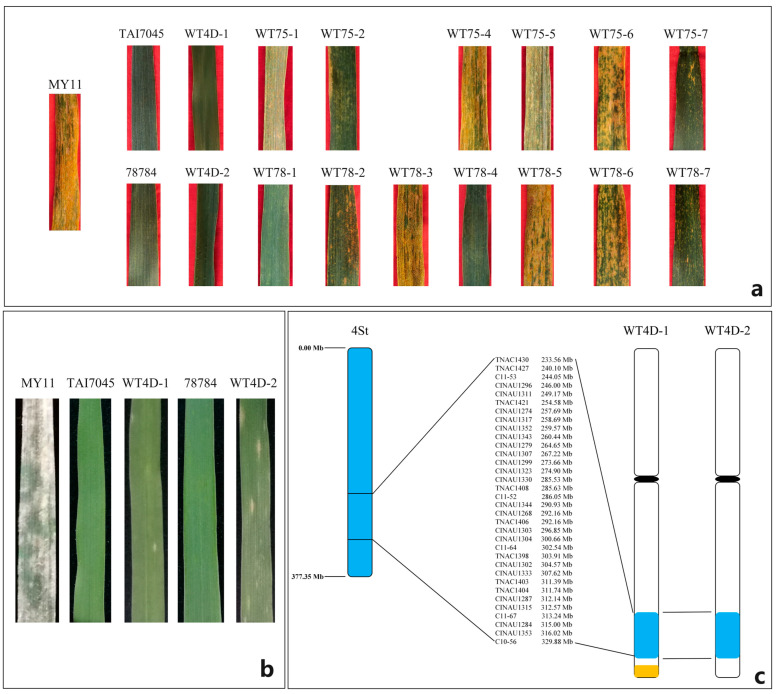
Physical location of wheat-*Th. intermedium* lines using rust resistance survey and molecular marker map. Stripe rust response (**a**) and powdery mildew response (**b**). The 34 4St-specific markers were screened and blasted to located on the 4StL of *Th. intermedium* genome sequence of V3.1 (**c**). The chromosome fragments with blue and orange backgrounds represent the introgression of chromosomes 4St and 4J, respectively.

Subsequently, we analyzed the *Th. intermedium*-specific amplification patterns of the DNA from TAI7045, 78784, WT4D-1 and WT4D-2. A total of 41 markers produced the 4St-specific polymorphic bands in WT4D-1, WT4D-2, TAI7045, 78784 and WT78-4 (4St addition line) (Table S1). Of them, 34 markers could be located into the physical intervals 233.56–329.88 Mb of the chromosome 4St by blasting the reference genomic sequences *Thinopyrum intermedium* V3.1 (https://phytozome-next.jgi.doe.gov/info/Tintermedium_v3_1, accessed on 12 April 2024) (Figure 6c). Above all, the PCR of marker C10-56 produced a 4St-specific fragment size of 120 bp (Figure 4). The physical location of these 4St-specific markers indicated that the C10-56 may be closed to the breakpoint of 4St insertion (Figure 5 and Figure 6c). One pair of TNAC1391 primers generated 4J-specific bands in WT4D-1 and TAI7045, but not in WT4D-2 and 78784, which was located on the distal part of the long arm of the chromosome 4J (487.81 Mb). The ND-FISH results and molecular marker surveys indicated that the wheat-*Th. intermedium* translocation chromosomes WT4D-1 and WT4D-2 were 4DS.4DL-4StL-4DL-4JL and 4DS.4DL-4StL-4DL, respectively.

### 2.5. Powdery Mildew and Stripe Rust Reactions for the Wheat-Th. intermedium Lines

The wheat-*Th. intermedium* lines and their parents were inoculated with *Pst* races CYR32, CYR33, and CYR34 at the adult plant stage in the field. The wheat parent MY11 was highly susceptible to all of these stripe rust pathotypes, and its infection type (IT) was scored as 4, while two wheat-*Th. intermedium* partial amphiploids TAI7045 and 78784 were highly immune to the mixture of *Pst* races (IT = 0) (Figure 6a). The difference between two wheat-*Th. intermedium* introgression lines, 1St-J^S^ (TAI7045) and 1St-J^S^ (78784), in stripe rust resistance was scored, which may be caused by the structure of two chromosomes in the end of the long arm (Figure S1). The plants carrying 3St, 4J, 5J.St, 5St or 6J^S^.J had higher ITs (3 or 4) (Table 1). Plants with 2St, 2St-J^S^, 4St and 7J^S^ chromosomes displayed high resistance for all *Pst* races tested (IT = 0–1). Noticeably, the recombinant lines WT4D-1 and WT4D-2 showed high *Pst* resistances as TAI70445 and 78784, indicating that the *Yr* locus originated from the 4St chromosome segment.

The lines WT4D-1 and WT4D-2 and their parents were further evaluated for resistance to powdery mildew in the greenhouse. The seedling ITs to powdery mildew were scored when the susceptible control MY11 was fully infected (IT = 4). In this stage, the lines WT4D-1 and WT4D-2 were susceptible to *Bgt* isolates with infection types 3–4. However, both of them displayed fully resistance (IT = 0;) from the five-leaf stage, indicating that they may have adult plant resistance (APR) (Figure 6b, Table 1). These results suggest that the *Th. intermedium* chromosome 4StL carries an excellent gene makeup for powdery mildew and stripe rust resistance and may be physically localized in the region 233.56–329.88 Mb. The chromosomal location of the resistance gene(s) will be verified using linkage analysis in future studies.

## 3. Discussion

Transferring desirable genes from wild relatives by interspecific hybridization is an important approach for broadening the genetic diversity of wheat [31,32]. A great number of wheat-alien species derivatives have been developed via distant hybridizations and chromosome engineering in the past few decades. However, the identification efficiency is relatively low in earlier research due to the complexity of the genomic composition, such as the allohexaploid species *Th. intermedium* [13,15,16,33,34]. Combining Oligo-FISH painting and ND-FISH using multiple oligo probes, we established an efficient system for precisely distinguishing *Th. intermedium* chromosome segments from wheat [27]. Based on this system, we screened a set of wheat-*Th. intermedium* addition lines from two hybrid populations, TAI7045/MY11 and 78784/MY11, in F_3_ generation. Although some introgressed complete *Th. intermedium* chromosomes carrying excellent genes, involving 2St, 3St, 4St, 4J and 7J^S^, have been reported in previous studies, they have different origins [16,23,35,36,37]. Han et al. [34] and Hu et al. [36] re-characterized a wheat-*Th. intermedium* addition line Z2 and found that it contained a substitution of one pair of 2D chromosomes by a pair of *Th. intermedium* chromosomes 2St-J^S^. In this study, five new wheat-*Th. intermedium* derivatives, including 1St-J^S^ (TAI7045), 1St-J^S^ (78784), 5St, 5J.St and 6J^S^.J, were found to cover several segments of the *Th. intermedium* genome, which can be important bridge materials for the excavation of *Th. intermedium* genetic resources.

Previous studies have suggested that the introduction of alien chromosomes may lead to structural variations of common wheat chromosomes [38,39]. Cui et al. [40] identified different types of chromosomal structural variation from three octoploid *Trititrigia* accessions (TE261-1, TE266-1, and TE346-1), which occurred in the chromosomes 1A, 6A, 6B, 2D and 7D. In the present study, two types of wheat recombinant chromosomes involving chromosomes 1A and 2D were also observed in TAI7045 (Figure 1), and a number of wheat chromosomal rearrangement events were detected in two hybrid populations (Figure S5). In addition, we also found two novel wheat-*Th. intermedium* insertional translocation chromosomes, T4DS.4DL-4StL-4DL and T4DS.4DL-4StL-4DL-4JL, which could be characterized by sequential ND-FISH with four newly developed probes and molecular marker analysis. We found that four markers including C10-56 were previously located in 4J by genetic mapping and comparison to the assembly of *Th. intermedium* v1.0 [30]; however, they were mapped on 4St by blasting for the updated *Th. intermedium* genome v3.1 and confirmed by PCR analysis in the present study (Figure 4, Table S1). It is thus pertinent to note that the combination of bioinformatic, cytogenetic and molecular analysis would be useful for precise identification of wheat-*Thinopyrum* introgression lines. The translocated chromosome T4DS.4DL-4StL-4DL from TAI7045 and 78784 has been transferred into common wheat individually. Another translocated chromosome was generated via the homoeologous chromosomal recombination between the chromosomes T4DS.4DL-4StL-4DL and 4J in the process of TAI7045/MY11. This may be due to a close relationship between *Th. intermedium* and *Aegilops tauschii*, and the chromosomes of linkage group 4 in wheat and *Th. intermedium* are highly prone to recombination [16,25,41].

The homoeologous group-4 chromosomes of the *Thinopyrum* species possess many desirable sources, such as a perennial habit, blue-grained characteristic, and resistance to stripe rust, powdery mildew, eyespot (*Tapesia yallundae*), etc. [42,43,44]. For example, Li et al. [45] found that the *Th. intermedium* chromosome 4Ai#2S (originating from *Ag. intermedium*, the Max Plank Institute in Germany) carries the eyespot-resistant gene(s). The 4Ai#2S.4DL translocation lines displayed superior resistance to WSMV (Wheat Streak Mosaic Virus) [46,47]. Two putative *Th. intermedium*-derived resistance genes, *Yr50* and *pmCH89*, were mapped on wheat chromosome arm 4BL [18,22]. Li et al. [16] and Li et al. [25] located the resistance gene(s) to stripe rust on FL0.60-1.00 of the 4J^S^S and on FL0-0.60 of the 4J^S^L, respectively. Gong et al. [48] developed a new wheat-*Th. scirpeum* 4E (4D) chromosomal substitution line (K16-730-3) that is resistant to stripe rust and powdery mildew. In the present study, we identified two stable wheat-*Th. intermedium* small segmental translocation lines, WT4D-1 and WT4D-2, and found that they are highly resistant to stripe rust and powdery mildew at the adult stage. Combining data from cytogenetic analysis and specific marker amplification, the resistant gene(s) for the two fungi was mapped in the 233.56-329.88 Mb region of 4StL. However, Li et al. [16] observed that the addition line L4 with a 4Ai#1 (originating from *Ag. intermedium* accession no. 75) chromosome (St-genome) was susceptible to stripe rust. This phenomenon is probably due to the chromosome 4St originating from different *Th. intermedium* subspecies, indicating that the exploitation of diversified wheat-*Th. intermedium* introgression lines remains necessary.

## 4. Materials and Methods

### 4.1. Plant Materials

Wheat-*Th. intermedium* partial amphiploids TAI7045 and 78784 were kindly provided by Dr. Zhijian Chang, Shanxi Academy of Agricultural Sciences, China. Wheat lines Chinese Spring (CS) and Mianyang11 (MY11) and the nullisomic–tetrasomic (NT) lines of the cultivars CS [49] and X24C10 (4J/4B substitution) [16] are maintained by the Center for Informational Biology, School of Life Science and Technology, University of Electronic Science and Technology of China. Two crosses, TAI7045/MY11 and 78784/MY11, were created, and the F_2_ plants were used to detect the transmission of *Thinopyrum* chromosomes, and partial amphiploids of F_3_, originating from 133 F_2_ plants with one or two alien chromosomes, were used to screen the stable wheat-*Th. intermedium*-derived lines. Two wheat-*Th. intermedium* translocation lines, WT4D-1 and WT4D-2, were also identified in F_3_, and the selfing progenies (F_4_ and F_5_) were examined using cytogenetic analysis.

### 4.2. Oligonucleotide Probes Development

Four new oligonucleotide probes for ND-FISH were designed based on the recently reported *D. villosum* genome [50] and *Ae. speltoides* genome [51] sequences, referring to the method described by Lang et al. [52]. Six previously reported oligo probes including Oligo-pSc119.2-1 and Oligo-pTa535-1 [53], Oligo-D [54] and Oligo-B11 [55] were also used in this study for karyotype analysis. All oligo probe sequences are listed in Table 2. Oligonucleotide probes were labeled with 6-carboxyfluorescein (6-FAM) or 6-carboxytetramethylrhodamine (TAMRA), synthesized by Shanghai Invitrogen Biotechnology Co. Ltd. (Shanghai, China).

### 4.3. ND-FISH and Sequential ND-FISH

The chromosome spreads of materials were carried out according to the procedure of Han et al. [57]. ND-FISH by the synthesized probes was performed as described by Fu et al. [56] with some modifications. The slides were counter-stained with DAPI (4′,6-diamidino-2-phenylindole) and examined using an Olympus BX-51 microscope (Shinjuku, Tokyo, Japan). Microphotographs of ND-FISH chromosomes were captured using a cooled DP70 CCD camera. Sequential ND-FISH was conducted on the same slide with different probes following the description by Wang et al. [58]. All images were optimized using Adobe Photoshop CS6 software (Adobe Systems Incorporated, San Jose, CA, USA).

### 4.4. Molecular Marker Analysis

Genomic DNA was extracted from young leaves of CS, MY11, TAI7045, 78784 and derived lines using a sodium dodecyl sulfate (SDS) protocol [59]. PCR-based Landmark Unique Gene (PLUG) primers [29], the CINAU (Cytogenetics Institute, Nanjing Agricultural University, Nanjing, China) primers [30] and the *Th. intermedium* (Thi)-specific markers [31] were designed and used for the physical location in specific chromosomes. An integrated physical map of molecular markers (including oligo probes) was constructed by searching the database of Wheat Genome Assembly ref. v2.0 from https://urgi.versailles.inra.fr/download/iwgsc/IWGSC_RefSeq_Assemblies/v2.0/ (accessed on 12 April 2024) and *Thinopyrum intermedium* v3.1 DOE-JGI from https://phytozome-next.jgi.doe.gov/info/Tintermedium_v3_1 (accessed on 12 April 2024) as described by Lang et al. [35] and Yu et al. [26]. Polymerase chain reaction (PCR), restriction enzyme digestion of the amplified products and nondenaturing polyacrylamidegel electrophoresis (PAGE) were conducted according to the description by Yu et al. [26].

### 4.5. Powdery Mildew and Stripe Rust Response Observations

To survey the phenotype of lines to powdery mildew and stripe rust, we used local mixed *Bgt* isolates and a mixture of *Pst* races CYR32, CYR33 and CYR34, respectively. The inoculation of *Bgt* were carried out at the four-leaf stage in a greenhouse at 22 °C and photoperiod of 14 h of light per day, and the APR response was recorded at 20 days post-inoculation; refer to Mohler et al. [60]. Stripe rust reactions were observed at the heading and grain-filling stages in the field at the Xindu Experimental Station, Chengdu, China; refer to Li et al. [7]. The powdery-mildew- and stripe-rust-susceptible cultivar Mianyang 11 (MY11) was examined as a control. Resistance testing was carried out on the F_3_ generation, with ten plants assessed for each of the lines. ITs were scored according to the system described by Bariana and McIntosh [61].

## Figures and Tables

**Table 1:** Responses of tested materials to stripe rust (*Pst*) and powdery mildew (*Bgt*) at the adult stage.

Material Name	The Added Alien Chromosome or the Wheat-*Th. intermedium* Recombinant Chromosome	ITs for *Pst*	ITs for *Bgt*
MY11	/	4	4
TAI7045	1St-J^S^, 2St, 3J^S^, 4J, 5J.St, 6J^S^.J, 7J^S^	0	0
WT75-1	1St-J^S^	4	nt
WT75-2	2St	;1	0
WT75-4	4J	3	3
WT75-5	5J.St	4	4
WT75-6	6J^S^.J	3	nt
WT75-7	7J^S^	;	3
WT4D-1	4DS.4DL-4StL-4DL-4JL	0	0;
78784	1St-J^S^, 2St.J^S^, 3St, 4St, 5St, 6J^S^.J, 7J^S^	0	0
WT78-1	1St-J^S^	;	nt
WT78-2	2St.J^S^	;1	1
WT78-3	3St	4	4
WT78-4	4St	0	0
WT78-5	5St	4	nt
WT78-6	6J^S^.J	3	nt
WT78-7	7J^S^	;	3
WT4D-2	4DS.4DL-4StL-4DL	0	0

“/” indicates that the line did not carry the alien chromosome. “nt” means not tested. ITs of “0” mean immune (no symptoms), “;” means near to immune (necrotic or chlorotic blotches without sporulation), 1–2 indicate resistance (necrotic or chlorotic blotches with only a trace of slight sporulation) and 3–4 indicate susceptible (moderate to abundant sporulation, with or without chlorosis or necrosis). “0;” means “0” or “;” and “;1” means “;” or “1”.

**Table 2:** Sequences of synthesized oligonucleotide probes.

Name of Probes	Nucleotide Sequences of Probes (5′-3′)	Length of Oligo Probes (bp)	Reference
Oligo-Dv86	GTCGTCGCTACCGCGACGACGTCCGCCTCGACTCGCGTTACCCTAAGAC	49	/
Oligo-7v108	TATTAACGTGGATAATCGAAATACTGAATTTTAGTATT	38	/
Oligo-v03-86	CGAGGCGGACGTCGTCGCGGTAGCGACGACGGACGCCGAGACGAGCACGT	50	/
Oligo-Ae369	GAAAGAATCCTTTGAAGCATCTGGTCGTCACAAACGTTTTGACTACT	47	/
Oligo-pSc119.2-1	CCGTTTTGTGGACTATTACTCACCGCTTTGGGGTCCCATAGCTAT	45	[53]
Oligo-pTa535-1	GACGAGAACTCATCTGTTACATGGGCACTTCAATGTTTTTTAAACTTATTTGAACTCCA	59	[53]
Oligo-B11	TCCGCTCACCTTGATGACAACATCAGGTGGAATTCCGTTCGAGGG	45	[54]
Oligo-D	TACGGGTGCCAAACGAGTGTCTGAAAGACTCCTCGAGAGGAAAATGCGAA	50	[55]
Oligo-pTa71-2	GGGCAAAACCACGTACGTGGCACACGCCGCGTA	33	[53]
Oligo-pSc200	CTCACTTGCTTTGAGAGTCTCGATCAATTCGGACTCTAGGTTGATTTTTGTATTTTCT	58	[56]

“/” refers to the newly developed probes in this study.

## Data Availability

Data are contained within the article or Supplementary Materials.

## Supplementary Materials

The following supporting information can be downloaded at https://www.mdpi.com/article/10.3390/plants13162333/s1: Figure S1: The karyotype analysis for the *Th. intermedium* chromosomes of TAI7045 (left) and 78784 (right) with probes Oligo-pSc119.2-1, Oligo-pTa535-1, Oligo-pDb12H, Oligo-pTa71-2 and Oligo-pSc200. Figure S2: Non-denaturing FISH of the common wheat MY11. The probes for sequential ND-FISH were Oligo-pSc119.2-1 (green) + Oligo-pTa535-1 (red) (a), Oligo-7v108 (green) + Dv86 (red) (b), Oligo-v03-86 (green) + Ae369 (red) (c), Oligo-v03-86 (green) (d), and Ae369 (red) (e). The 4D chromosomes are shown (f). Bars represent 10 μm. Figure S3: ND-FISH of the plants of F_2_ progenies from the two crosses TAI7045/MY11 (a–d) and 78784/MY11 (e–h) with the probes Oligo-pSc119.2-1 (green), Oligo-pTa535-1 (red), Oligo-pTa71-2 (red) and Oligo-pSc200 (green). The plants TAI7045-MY11-237 (2n = 44) (a), TAI7045-MY11-200 (2n = 48) (b), TAI7045-MY11-130 (2n = 50) (c), TAI7045-MY11-232 (2n = 53) (d), 78784-MY11-178 (2n = 43) (e), 78784-MY11-241 (2n = 45) (f), 78784-MY11-311 (2n = 47) (f), and 78784-MY11-205 (2n = 51) (h) were selected for displaying the variation in chromosome numbers and the transmission of *Thinopyrum* chromosomes. Bars represent 10 μm. Figure S4: Cytogenetic characterization of *Thinopyrum* chromosome introgression lines from F_3_ generations of the cross TAI7045/MY11. The introgressed *Thinopyrum* chromosomes 1St-J^S^ (a,b), 2St (c,d), 4J (e), 5J.St (f), 6J^S^.J (g), and 7J^S^ (h). Yellow arrows and text notes indicate the *Thinopyrum* chromosomes. Bars represent 10 μm. Figure S5: Cytogenetic characterisation of *Thinopyrum* chromosome introgression lines from F_3_ generations of the cross 78784/MY11. The introgressed *Thinopyrum* chromosomes 1St-J^S^ (a,b), 2St-J^S^ (c,d), 3St (e,f), 5St (g,h), 4St (i), 6J^S^.J (g), and 7J^S^ (h). Yellow arrows and text notes indicate the *Thinopyrum* chromosomes. Bars represent 10 μm. Table S1: Sequences of 42 pairs of *Thinopyrum intermedium* chromosome 4St-specific primers in WT4D-1 and WT4D-2.

## References

[1] International Wheat Genome Sequencing Consortium (IWGSC) (2014). A chromosome-based draft sequence of the hexaploid bread wheat (*Triticum aestivum*) genome. Science.

[2] Morgounov A., Tufan H.A., Sharma R., Akin B., Bagci A., Braun H.J., Kaya Y., Keser M., Payne T.S., Sonder K. (2012). Global incidence of wheat rusts and powdery mildew during 1969–2010 and durability of resistance of winter wheat variety bezostaya 1. Eur. J. Plant Pathol..

[3] Chen W.Q., Wellings C., Chen X.M., Kang Z.S., Liu T.G. (2014). Wheat stripe (yellow) rust caused by *Puccinia striiformis* f. sp. *tritici*. Mol. Plant Pathol..

[4] Savary S., Willocquet L., Pethybridge S.J., Esker P., McRoberts N., Nelson A. (2019). The global burden of pathogens and pests on major food crops. Nat. Ecol. Evol..

[5] McIntosh R.A., Dubcovsky J., Rogers W.J., Xia X.C., Raupp W.J. (2018). Catalogue of gene symbols for wheat. Ann. Wheat Newsl..

[6] Tan C., Li G., Cowger C., Carver B.F., Xu X. (2019). Characterization of *Pm63*, a powdery mildew resistance gene in Iranian landrace PI 628024. Theor. Appl. Genet..

[7] Li G.R., Tang L.R., Yin Y., Zhang A.H., Yu Z.H., Yang E.N., Tang Z.X., Fu S.L., Yang Z.J. (2020). Molecular dissection of *Secale africanum* chromosome 6^Rafr^ in wheat enabled localization of genes for resistance to powdery mildew and stripe rust. BMC Plant Biol..

[8] Baker L., Grewal S., Yang C.Y., Hubbart-Edwards S., Scholefield D., Ashling S., Burridge A.J., Przewieslik-Allen A.M., Wilkinson P.A., King I.P. (2020). Exploiting the genome of *Thinopyrum elongatum* to expand the gene pool of hexaploid wheat. Theor. Appl. Genet..

[9] Liu C., Han R., Wang X.L., Gong W.P., Cheng D.G., Cao X.Y., Liu A.F., Li H.S., Liu J.J. (2020). Research progress of wheat wild hybridization, disease resistance genes transfer and utilization. Sci. Agric. Sin..

[10] Wan A.M., Zhao Z.H., Chen X.M., He Z.H., Jin S.L., Jia Q.Z., Yao G., Yang J.X., Wang B.T., Li G.B. (2004). Wheat stripe rust epidemic and virulence of *Puccinia striiformis* f. sp. *tritici* in China in 2002. Plant Dis..

[11] Zhang H.T., Guan H.Y., Li J.T., Zhu J., Xie C.J., Zhou Y.L., Duan X.Y., Yang T., Sun Q.X., Liu Z.Y. (2010). Genetic and comparative genomics mapping reveals that a powdery mildew resistance gene Ml3D232 originating from wild emmer co-segregates with an NBS-LRR analog in common wheat (*Triticum aestivum* L.). Theor. Appl. Genet..

[12] Tsvelev N.N., Fedorov A.A. (1983). Grasses of the Soviet Union.

[13] Bao Y.G., Wu X., Zhang C., Li X.F., He F., Qi X.L., Wang H.G. (2014). Chromosomal constitutions and reactions to powdery mildew and stripe rust of four novel wheat-*Thinopyrum intermedium* partial amphiploids. J. Genet. Genom..

[14] Zhang X., Cui C.H., Bao Y.G., Wang H.G., Li X.F. (2020). Molecular cytogenetic characterization of a novel wheat-*Thinopyrum intermedium* introgression line tolerant to phosphorus deficiency. Crop J..

[15] Chen Q., Conner R.L., Laroche A., Ji W., Armstrong K.C., Fedak G. (1999). Genomic in situ hybridization analysis of *Thinopyrum* chromatin in a wheat-*Th. intermedium* partial amphiploid and six derived chromosome addition lines. Genome.

[16] Li J.B., Lang T., Li B., Yu Z.H., Wang H.J., Li G., Yang E.N., Yang Z.J. (2017). Introduction of *Thinopyrum intermedium* ssp. *trichophorum* chromosomes to wheat by trigeneric hybridization involving *Triticum*, *Secale* and *Thinopyrum* genera. Planta.

[17] Fedak G., Chen Q., Conner R.L., Laroche A., Petroski R., Armstrong K.W. (2000). Characterization of wheat-*Thinopyrum* partial amphiploids by meiotic analysis and genomic in situ hybridization. Genome.

[18] Hou L.Y., Zhang X.J., Li X., Jia J.Q., Yang H.Z., Zhan H.X., Qiao L.Y., Guo H.J., Chang Z.J. (2015). Mapping of powdery mildew resistance gene *pmCH89* in a putative wheat-*Thinopyrum intermedium* introgression line. Int. J. Mol. Sci..

[19] Yang G.T., Zhang N., Boshoff W.H.P., Li H.W., Li B., Li Z.S., Zheng Q. (2023). Identification and introgression of a novel leaf rust resistance gene from *Thinopyrum intermedium* chromosome 7J^S^ into wheat. Theor. Appl. Genet..

[20] Luo P.G., Luo H.Y., Chang Z.J., Zhang H.Y., Zhang M., Ren Z.L. (2009). Characterization and chromosomal location of *Pm40* in common wheat: A new gene for resistance to powdery mildew derived from *Elytrigia intermedium*. Theor. Appl. Genet..

[21] He R.L., Chang Z.J., Yang Z.J., Zhan H.X., Zhang X.J., Liu J.X. (2009). Inheritance and mapping of powdery mildew resistance gene *Pm43* introgressed from *Thinopyrum intermedium* into wheat. Theor. Appl. Genet..

[22] Liu J., Chang Z.J., Zhang X.J., Yang Z.J., Li X., Jia J.Q., Zhan H.X., Guo H.J., Wang J.M. (2013). Putative *Thinopyrum intermedium*-derived stripe rust resistance gene *Yr50* maps on wheat chromosome arm 4BL. Theor. Appl. Genet..

[23] Wang S.W., Wang C.Y., Feng X.B., Zhao J.X., Deng P.C., Wang Y.J., Zhang H., Liu X.L., Li T.D., Chen C.H. (2022). Molecular cytogenetics and development of St-chromosome-specific molecular markers of novel stripe rust resistant wheat-*Thinopyrum intermedium* and wheat-*Thinopyrum ponticum* substitution lines. BMC Plant Biol..

[24] Zhang X.J., Li J.B., Ge Y.D., Guan H.X., Li G.R., Zhang S.W., Wang X.L., Li X., Chang Z.J., Zhang P. (2022). Molecular cytogenetic characterization of a new wheat-*Thinopyrum intermedium* homoeologous group-6 chromosome disomic substitution line with resistance to leaf rust and stripe rust. Front. Plant Sci..

[25] Li G.R., Chen Q.H., Jiang W.X., Zhang A.H., Yang E.N., Yang Z.J. (2022). Molecular and cytogenetic identification of wheat-*Thinopyrum intermedium* double substitution line-derived progenies for stripe rust resistance. Plants.

[26] Yu Z.H., Wang H.J., Xu Y.F., Li Y.S., Lang T., Yang Z.J., Li G.R. (2019). Characterization of chromosomal rearrangement in new wheat-*Thinopyrum intermedium* addition lines carrying *Thinopyrum*-specific grain hardness genes. Agronomy.

[27] Yu Z.H., Wang H.J., Yang E.N., Li G.R., Yang Z.J. (2022). Precise identification of chromosome constitution and rearrangements in wheat–*Thinopyrum intermedium* derivatives by ND-FISH and Oligo-FISH painting. Plants.

[28] Ishikawa G., Nakamura T., Ashida T., Saito M., Nasuda S., Endo T.R., Wu J.Z., Matsumoto T. (2009). Localization of anchor loci representing five hundred annotated rice genes to wheat chromosomes using PLUG markers. Theor. Appl. Genet..

[29] Wang H.Y., Dai K.L., Xiao J., Yuan C.X., Zhao R.H., Doležel J., Wu Y.F., Cao A.Z., Chen P.D., Zhang S.Z. (2017). Development of intron targeting (IT) markers specific for chromosome arm 4VS of *Haynaldia villosa* by chromosome sorting and next-generation sequencing. BMC Genom..

[30] Qiao L.Y., Liu S.J., Li J.B., Li S.J., Yu Z.H., Liu C., Li X., Liu J., Ren Y.K., Zhang P. (2021). Development of sequence-tagged site marker set for identification of J, J^S^, and St sub-genomes of *Thinopyrum intermedium* in wheat background. Front. Plant Sci..

[31] Chen Q., Conner R.L., Li H.J., Sun S.C., Ahmad F., Laroche A., Graf R.J. (2003). Molecular cytogenetic discrimination and reaction to wheat streak mosaic virus and the wheat curl mite in Zhong series of wheat-*Thinopyrum intermedium* partial amphiploids. Genome.

[32] Sepsi A., Molnár I., Szalay D., Molnár-Láng M. (2008). Characterization of a leaf rust-resistant wheat-*Thinopyrum ponticum* partial amphiploid BE-1, using sequential multicolor GISH and FISH. Theor. Appl. Genet..

[33] He M.Y., Xu Z.Y., Zou M.Q., Dawei Z., Zhensan P., Shui H. (1988). The establishment of two sets of alien addition lines of wheat-wheatgrass. Sci. China Ser. B.

[34] Han F.P., Fedak G., Benabdelmouna A., Armstrong K., Ouellet T. (2003). Characterization of six wheat x *Thinopyrum intermedium* derivatives by GISH, RFLP, and multicolor GISH. Genome.

[35] Lang T., La S.X., Li B., Yu Z.H., Chen Q.H., Li J.B., Yang E.N., Li G.R., Yang Z.J. (2018). Precise identification of wheat-*Thinopyrum intermedium* translocation chromosomes carrying resistance to wheat stripe rust in line Z4 and its derived progenies. Genome.

[36] Hu L.J., Li G.R., Zhan H.X., Liu C., Yang Z.J. (2012). New St-chromosome-specific molecular markers for identifying wheat-*Thinopyrum intermedium* derivative lines. J. Genet..

[37] Nie L.M., Yang Y.N., Zhang J., Fu T.H. (2019). Disomic chromosome addition from *Thinopyrum intermedium* to bread wheat appears to confer stripe rust resistance. Euphytica.

[38] Rey E., Abrouk M., Keeble-Gagnère G., Karafiátová M., Vrána J., Balzergue S., Soubigou-Taconnat L., Brunaud V., Martin-Magniette M.L., Endo T.R. (2018). Transcriptome reprogramming due to the introduction of a barley telosome into bread wheat affects more barley genes than wheat. Plant Biotechnol. J..

[39] Wang Y.Z., Cao Q., Zhang J.J., Wang S.W., Chen C.H., Wang C.Y., Zhang H., Wang Y.J., Ji W.Q. (2020). Cytogenetic analysis and molecular marker development for a new wheat-*Thinopyrum ponticum* 1J^S^ (1D) disomic substitution line with resistance to stripe rust and powdery mildew. Front. Plant Sci..

[40] Cui Y., Xing P.Y., Qi X.L., Bao Y.G., Wang H.G., Wang R.R., Li X.F. (2021). Characterization of chromosome constitution in three wheat-*Thinopyrum intermedium* amphiploids revealed frequent rearrangement of alien and wheat chromosomes. BMC Plant Biol..

[41] Mahelka V., Kopecký D., Paštová L. (2011). On the genome constitution and evolution of intermediate wheatgrass (*Thinopyrum intermedium*: Poaceae, Triticeae). BMC Evol. Biol..

[42] Lammer D., Cai X.W., Arterburn M., Chatelain J., Murray T., Jones S. (2004). A single chromosome addition from *Thinopyrum elongatum* confers a polycarpic, perennial habit to annual wheat. J. Exp. Bot..

[43] Li Z.S., Mu S.M., Zhou H.P., Wu J. (1986). The establishment and application of blue-grained monosomics in wheat chromosome engineering. Cereal Res. Commun..

[44] Yang G.T., Deng P.C., Ji W.Q., Fu S.L., Li H.W., Li B., Li Z.S., Zheng Q. (2023). Physical mapping of a new powdery mildew resistance locus from *Thinopyrum ponticum* chromosome 4AgS. Front. Plant Sci..

[45] Li H.J., Arterburn M., Jones S.S., Murray T.D. (2005). Resistance to eyespot of wheat, caused by *Tapesia yallundae*, derived from *Thinopyrum intermedium* homoeologous group 4 chromosome. Theor. Appl. Genet..

[46] Wang R.R., Zhang X.Y. (1996). Characterization of the translocated chromosome using fluorescence in situ hybridization and random amplified polymorphic DNA on two *Triticum aestivum*-*Thinopyrum intermedium* translocation lines resistant to wheat streak mosaic or barley yellow dwarf virus. Chromosome Res..

[47] Ali N., Heslop-Harrison J.P., Ahmad H., Graybosch R.A., Hein G.L., Schwarzacher T. (2016). Introgression of chromosome segments from multiple alien species in wheat breeding lines with wheat streak mosaic virus resistance. Heredity.

[48] Gong B., Zhang H., Yang Y.L., Zhang J.W., Zhu W., Xu L.L., Wang Y., Zeng J., Fan X., Sha L. (2022). Development and identification of a novel wheat-*Thinopyrum scirpeum* 4E (4D) chromosomal substitution line with stripe rust and powdery mildew resistance. Plant Dis..

[49] Sears E.R., Riley R., Lewis K.R. (1966). Nullisomic-tetrasomic combinations in hexaploid wheat. Chromosome Manipulations and Plant Genetics.

[50] Zhang X., Wang H.Y., Sun H.J., Li Y.B., Feng Y.L., Jiao C.Z., Li M.L., Song X.Y., Wang T., Wang Z.K. (2023). A chromosome-scale genome assembly of *Dasypyrum villosum* provides insights into its application as a broad-spectrum disease resistance resource for wheat improvement. Mol. Plant.

[51] Wang X.F., Zhang Y.X., Niu Y.Q., Sha Y., Wang Z.H., Zhang Z.B., Yang J., Liu B., Li L.F. (2023). Post-hybridization introgression and natural selection promoted genomic divergence of *Aegilops speltoides* and the four S*-genome diploid species. Plant J..

[52] Lang T., Li G.R., Wang H.J., Yu Z.H., Chen Q.H., Yang E.N., Fu S.L., Tang Z.X., Yang Z.J. (2019). Physical location of tandem repeats in the wheat genome and application for chromosome identification. Planta.

[53] Tang Z.X., Yang Z.J., Fu S.L. (2014). Oligonucleotides replacing the roles of repetitive sequences pAs1, pSc119.2, pTa-535, pTa71, CCS1, and pAWRC.1 for FISH analysis. J. Appl. Genet..

[54] Xi W., Tang Z.X., Tang S.Y., Yang Z.J., Luo J., Fu S.L. (2019). New ND-FISH-positive Oligo probes for identifying *Thinopyrum* Chromosomes in wheat backgrounds. Int. J. Mol. Sci..

[55] Tang S.Y., Tang Z.X., Qiu L., Yang Z.J., Li G.R., Lang T., Zhu W.Q., Zhang J.H., Fu S.L. (2018). Developing new oligo probes to distinguish specific chromosomal segments and the A, B, D genomes of wheat (*Triticum aestivum* L.) using ND-FISH. Front. Plant Sci..

[56] Fu S.L., Chen L., Wang Y.Y., Li M., Yang Z.J., Qiu L., Yan B.J., Ren Z.L., Tang Z.X. (2015). Oligonucleotide probes for ND-FISH analysis to identify rye and wheat chromosomes. Sci. Rep..

[57] Han F.P., Lamb J.C., Birchler J.A. (2006). High frequency of centromere inactivation resulting in stable dicentric chromosomes of maize. Proc. Natl. Acad. Sci. USA.

[58] Wang H.J., Yu Z.H., Li G.R., Yang Z.J. (2019). Diversified chromosome rearrangements detected in a wheat–*Dasypyrum breviaristatum* substitution line induced by gamma-ray irradiation. Plants.

[59] Liu C., Li G.R., Sehgal S.K., Jia J.Q., Yang Z.J., Friebe B., Gill B.S. (2010). Genome relationships in the genus *Dasypyrum*: Evidence from molecular phylogenetic analysis and in situ hybridization. Plant Syst. Evol..

[60] Mohler V., Stadlmeier M. (2019). Dynamic QTL for adult plant resistance to powdery mildew in common wheat (*Triticum aestivum* L.). J. Appl. Genet..

[61] Bariana H.S., McIntosh R.A. (1993). Cytogenetic studies in wheat. 15. Location of rust resistance genes in VPM1 and their genetic linkage with other disease resistance genes in chromosome 2A. Genome.

