# Supplementary material for: Characterization of New Wheat-Thinopyrum intermedium Derivative Lines with Superior Genes for Stripe Rust and Powdery Mildew Resistance
Source: Plants (Basel). 2024 Aug 22;13(16):2333. doi: 10.3390/plants13162333 (PMC11359552; doi:10.3390/plants13162333)
Supplement: Supplementary file 1 [file plants-13-02333-s001.zip › plants-3006389-supplementary.pdf]

Supplementary Materials

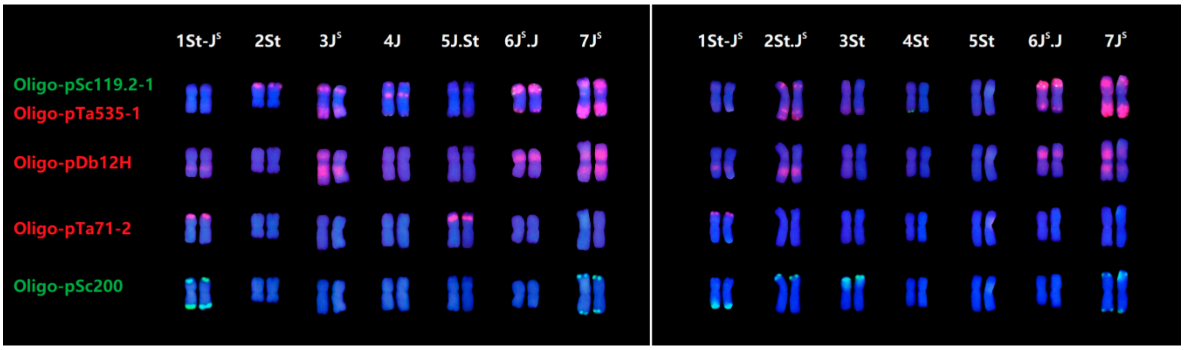

Figure S1. The karyotype analysis for the *Th. intermedium* chromosomes of TAI7045 (left) and 78784 (right) with probes Oligo-pSc119.2-1, Oligo-pTa535-1, Oligo-pDb12H, Oligo-pTa71-2 and Oligo-pSc200.

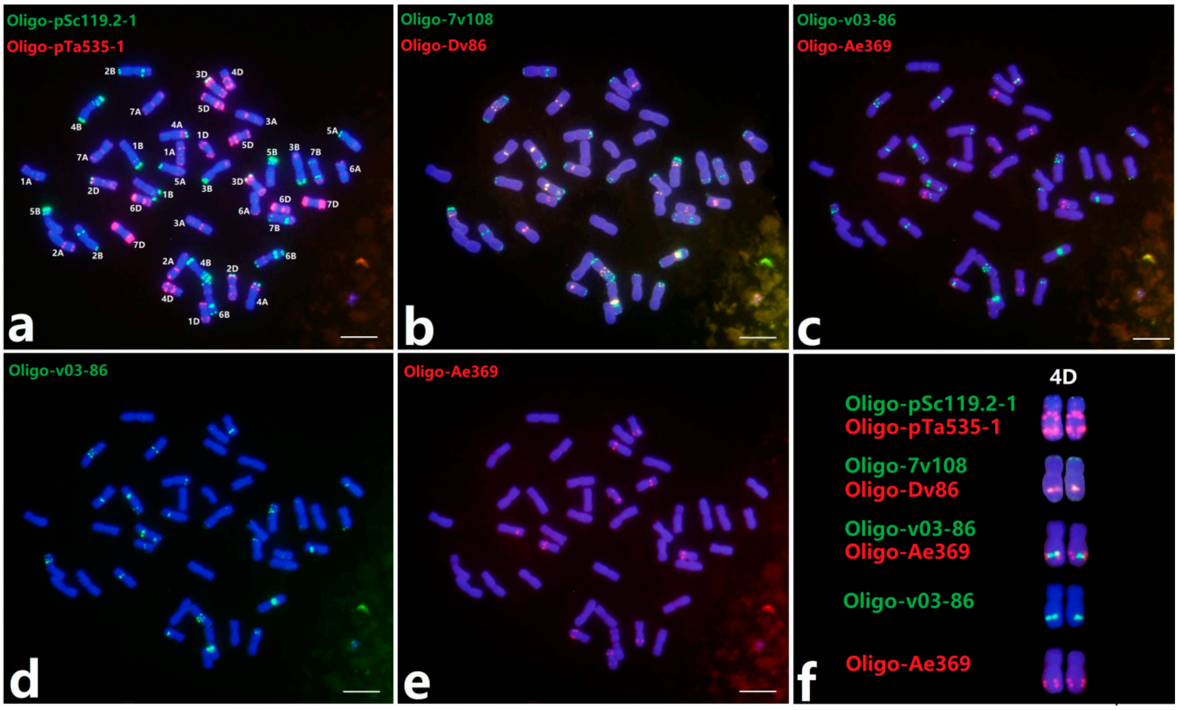

Figure S2. Non-denaturing FISH of the common wheat MY11. The probes for sequential ND-FISH were Oligo-pSc119.2-1 (green) + Oligo-pTa535-1 (red) (a), Oligo-7v108 (green) + Dv86 (red) (b), Oligo-v03-86 (green) + Ae369 (red) (c), Oligo-v03-86 (green) (d), and Ae369 (red) (e). 4D chromosomes are shown (f). Bars represent 10 μm.

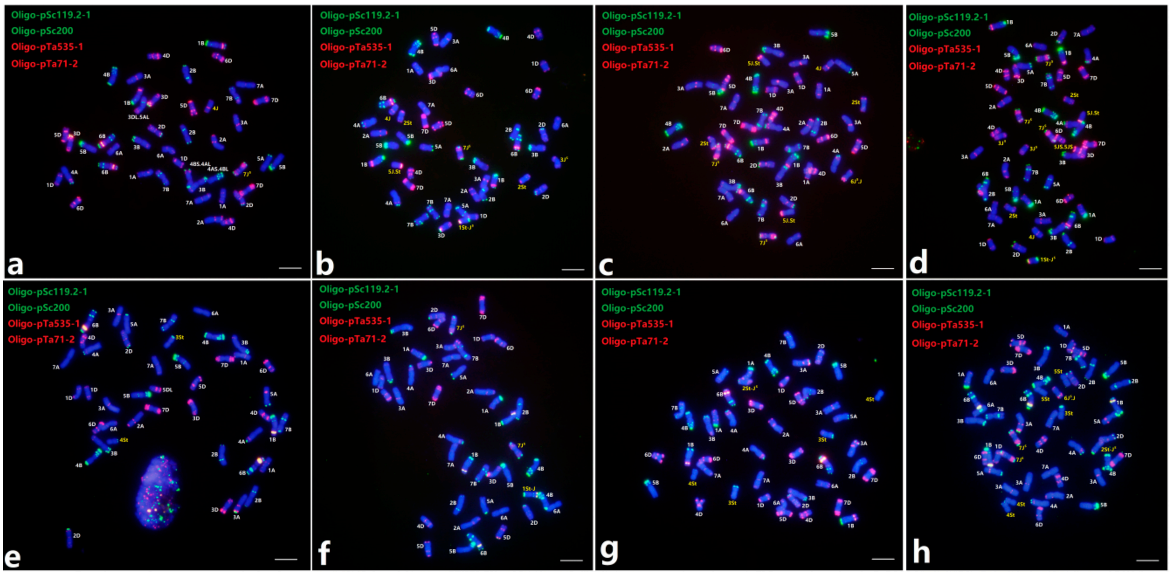

Figure S3. ND-FISH of the plants of F_2 progenies from the two crosses TAI7045/MY11 (a-d) and 78784/MY11 (e-h) with the probes Oligo-pSc119.2-1 (green), Oligo-pTa535-1 (red), Oligo-pTa71-2 (red) and Oligo-pSc200 (green). The plants, TAI7045-MY11-237 (2n = 44) (a), TAI7045-MY11-200 (2n = 48) (b), TAI7045-MY11-130 (2n = 50) (c), TAI7045-MY11-232 (2n = 53) (d), 78784-MY11-178 (2n = 43) (e), 78784-MY11-241 (2n = 45) (f), 78784-MY11-311 (2n = 47) (f), and 78784-MY11-205 (2n = 51) (h) were selected for displaying the variation of chromosome numbers and the transmission of *Thinopyrum* chromosomes. Bars represent 10 μm.

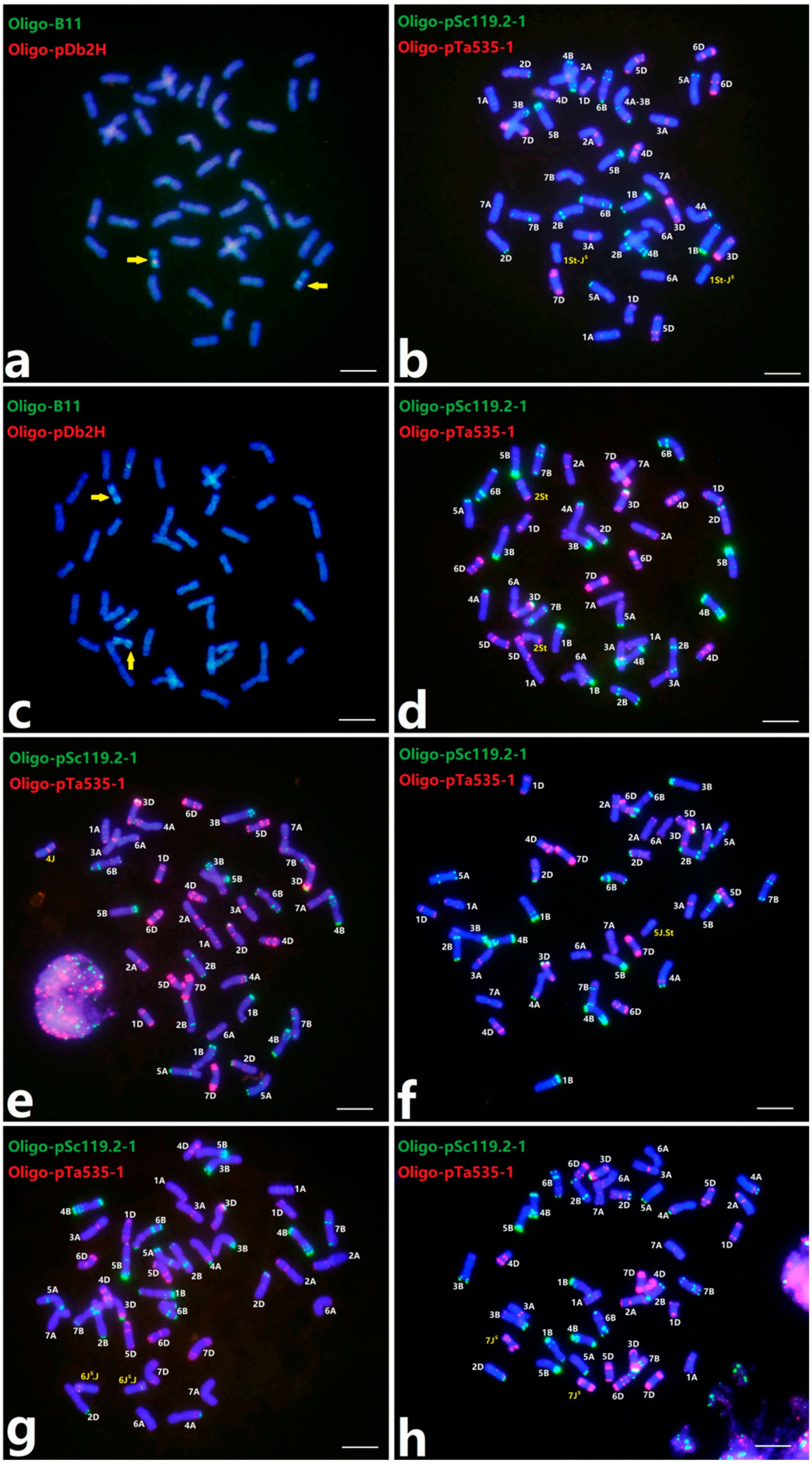

Figure S4. Cytogenetic characterisation of *Thinopyrum* chromosomes introgression lines from F3 generations of the cross TAI7045/MY11. The introgressed *Thinopyrum* chromosomes, 1St-J^S (a, b), 2St (c, d), 4J (e), 5J.St (f), 6J^S.J (g), and 7J^S (h). Yellow arrows and text notes indicated the *Thinopyrum* chromosomes. Bars represent 10 µm.

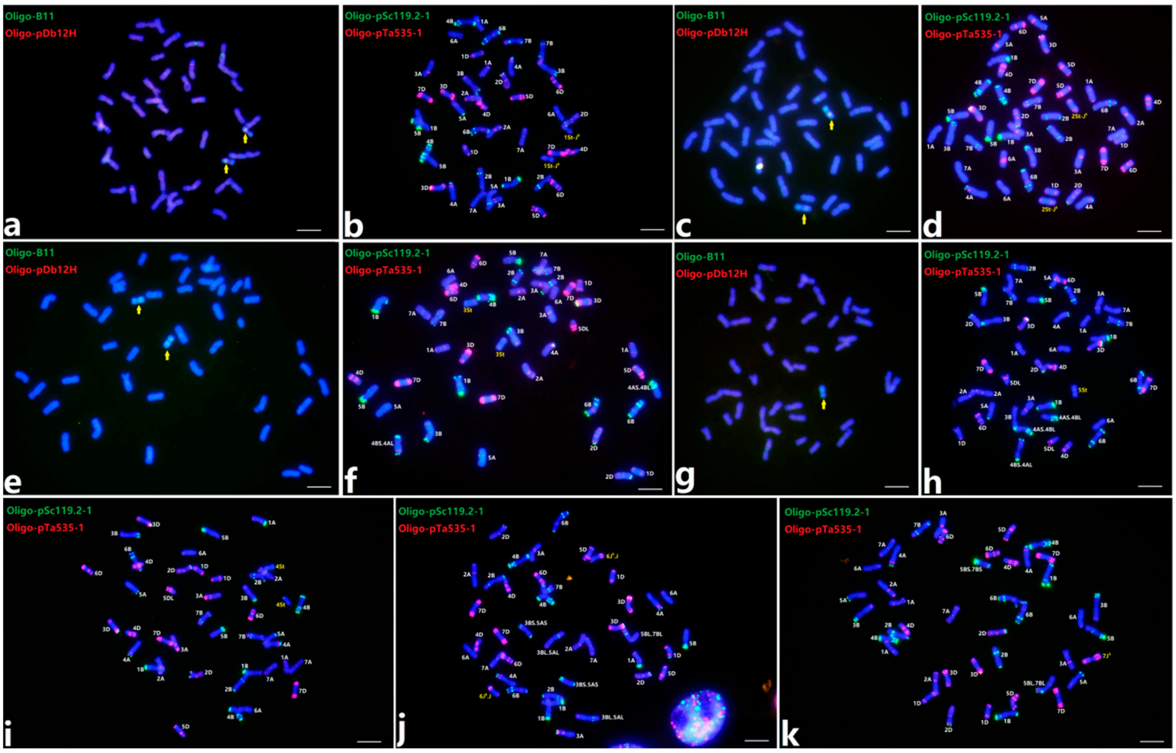

Figure S5. Cytogenetic characterisation of *Thinopyrum* chromosomes introgression lines from F_3 generations of the cross 78784/MY11. The introgressed *Thinopyrum* chromosomes, 1St-J^S (a, b), 2St-J^S (c, d), 3St (e, f), 5St (g, h), 4St (i), 6J^S.J (g), and 7J^S (h). Yellow arrows and text notes indicated the *Thinopyrum* chromosomes. Bars represent 10 µm.

Table S1 Sequences of 42 pairs of *Th. intermedium* chromosome 4St-specific primers in WT4D-1 and WT4D-2.

Primer name	Primer sequence (5'-3')	Physical location on 4St (Mb)	Restriction enzyme	Product size (bp)	Annealing temperature (°C)
TNAC1430	F: CAACGACAGCCCAACCAT	233.56	*Taq*I	680	60
	R: TTCTCATCCTCATAAACCACAG				
TNAC1427	F: AGGCCTGATATGCTTCATTGT	240.10	*Taq*I	290	60
	R: ATGGTGATGGAGCGGTTC				
C11-53	F: AGTTCCCGTGGAGAAAGAAC	244.05		400	60
	R: CCTCTGTTGGTACCTTGTGC				
CINAU1296	F: GGGTGGTGGTTGATGGCAA	246.00		500	60

	R: CCTTGTACGCCTTGCAGATG				
CINAU1311	F: CTTAACGCTCTGATGAAGGGA<br>R: AAGCACGTTCCTTCAGCAAG	249.17		300	60
TNAC1421	F: ATCCGCTTCTCCAAGTTCTTC<br>R: GTCCGATCCACTTCTTCAGGT	254.58	*Taq*I	1310	60
CINAU1274	F: GACATCATGCGCTGCTGG<br>R: GCGGCTGTTCCTTGATTGG	257.69		300	60
CINAU1317	F: CAGTTGGCCAAGAACCTAGC<br>R: GACGGGCGATGCTATCCA	258.69		700	60
CINAU1352	F: AACTCTGCGTTCACATTGGC<br>R: GATAGGGGAATCACATGGCC	259.57		300	60
CINAU1343	F: TGGAGATAGAGCGGGAGAAAC<br>R: AGCGAGCATCTTCAAGAGGA	260.44		1350	60
CINAU1279	F: AGTCAAACGCAGATTTCGTGA<br>R: CAGTCACCAATAACATGCCCA	264.65		740	60
CINAU1307	F: AGTTATTTCCTGGCCAGAAAACT<br>R: TTGGAATCAGCATCCAGGGA	267.22		430	60
CINAU1299	F: TGGCACTGGAACTCAACTGT<br>R: CAACGAGTGCAAGCAGGAAT	273.66		330	60
CINAU1323	F: GGGTGCTTTACTTTGTTGAGGT<br>R: CATGCGCCTCCTTGAGTG	274.90		700	60
CINAU1330	F: GTTCACCGTCATGCTGAGG<br>R: CTCCACCTTGCTCCTCTCC	285.53		680	60
TNAC1408	F: CAGGAAGTTGGTACCATTGTGA<br>R: CTTGCAGCCTCCTATTGATTC	285.63	*Taq*I	1350	60

C11-52	F: GATGACACCTGCTGGAACTC<br>R: ACGATAATTCCTGGCCTTTC	286.05		150	60
CINAU1344	F: CAGTGCCTCGACCTCCAA<br>R: CCTGTTCCAGCTCCTCTTCA	290.93		680	60
CINAU1268	F: AACCCTGTGAATTATGGCCG<br>R: GTTCTCCTGTCTCTTCCTCCC	292.16		380	60
TNAC1406	F: CCAAGAGTTCTGCACGTTGAT<br>R: GTCAGCTTTCCTGTGTGGAAG	292.16	*Taq*I	680	60
CINAU1303	F: AGAATGGCGCGAAGTAGAGA<br>R: GGCGGGGTTATCTTTGTCTTC	296.85		880	60
CINAU1304	F: CATCGGTCCCTACATTTGCG<br>R: CCGGAACATACTTCAGCCAG	300.66		280	60
C11-64	F: TTTTGGTAGCCTCGTGACAT<br>R: CATCGGCATCAATCAAAGAG	302.54		260	60
TNAC1398	F: CAAGGCAGGTGCTGATATTGT<br>R: ACCCAGGGTTGACTGACATAA	303.91	*Taq*I	850	60
CINAU1302	F: CTGAAAGGCGCATTTCTGGT<br>R: TCCTTGGCGAAGATCACAGT	304.57		950	60
CINAU1333	F: AGTGAGCCAGATATCCTTGACA<br>R: CCCAAGTTAGAGCATCTTCTGG	307.62		250	60
TNAC1403	F: CCTCCTCCATTGCGAGATAAC<br>R: GTAGTAACGCTGAAGGGTTCG	311.39	*Taq*I	200	60
TNAC1404	F: ACTCCATGATATTTGCCATGC<br>R: TGGAGAAATCGGTTTAGAGCA	311.74	*Taq*I	1100	60
CINAU1287	F: TGAACAATTACGACAAGTTCCT	312.14		280	60

	G				
	R: TGTCTGCAAGTTTACTGCCA				
CINAU1315	F: CAGCGGATTTCAGAGCTCAG	312.57		450	60
	R: AACTCGGCCAACCTGTCC				
C11-67	F: TGCTAGCTGCAGTTTCCCCA	313.24		200	60
	R: GCTCTCCTATCTACAACCGGGTC				
CINAU1284	F: TCCAAGACCATGTACCTCGAG	315.00		520	60
	R: GAACCTCTCCTGCCCAGC				
CINAU1353	F: TGGAGATCTATGCGGTGTTGT	316.02		290	60
	R: TTGACTGATGTGTAAGCTCCAA				
C10-56	F: CCCTGAAATGTAATCGCGGG	329.88		120	60
	R: CACAGTTACCTTTGCGAGTGT				
TNAC1419	F: CGAGCAACTGTTCAAGGAGAC	-	*Taq*I	750	60
	R: TGAAGCAGGACTTGTGGTAGG				
TNAC1442	F: CATTGGAACCACCTCCTCATC	-	*Taq*I	200	60
	R: AGCTTTGTAAGACGCAGCTCA				
TNAC1594	F: CCTCCAGAACAAGACCCAGAT	-	*Taq*I	300	60
	R: GCAATGACTCCCTCGAACAT				
TNAC1523	F: GGCGAGCACTTCAAGTCATTA	-	*Taq*I	420	60
	R: GTAGAGACGGCAGGTTTCGTT				
C10-45	F: CCAAACAGCATGCAACGCTA	-		110	60
	R: ACATCGCTCAAATGTTCCTGC				
C10-49	F: TGCCCTCAAATTTCAAACATACACA	-		125	60
	R: ACGGCAAATTGGGGGTTGA				
C10-54	F: CGACCTTGGTGGATTGGGTG	-		110	60

	R: GTCTATCCAATCAGGCCTCCC

notes: "-" means that the primer was not located on the chromosome 4St by blast.
